# Pannexin 2 Localizes at ER-Mitochondria Contact Sites

**DOI:** 10.3390/cancers11030343

**Published:** 2019-03-11

**Authors:** Maxence Le Vasseur, Vincent C. Chen, Kate Huang, Wayne A. Vogl, Christian C. Naus

**Affiliations:** 1 Department of Cellular and Physiological Sciences, The Life Sciences Institute, University of British Columbia, Vancouver, BC V6T 1Z3, Canada; levasseurmaxence@gmail.com (M.L.V.); ChenV@brandonu.ca (V.C.C.); katehuang925@gmail.com (K.H.); vogl@mail.ubc.ca (W.A.V.); 2 Department of Obstetrics and Gynaecology, University of British Columbia, Vancouver, BC V6T 1Z3, Canada

**Keywords:** pannexin 2, gap junction, mitochondria, endoplasmic reticulum, contact sites, mitochondria-associated membranes, apoptosis

## Abstract

Endomembrane specialization allows functional compartmentalization but imposes physical constraints to information flow within the cell. However, the evolution of an endomembrane system was associated with the emergence of contact sites facilitating communication between membrane-bound organelles. Contact sites between the endoplasmic reticulum (ER) and mitochondria are highly conserved in terms of their morphological features but show surprising molecular diversity within and across eukaryote species. ER-mitochondria contact sites are thought to regulate key processes in oncogenesis but their molecular composition remains poorly characterized in mammalian cells. In this study, we investigate the localization of pannexin 2 (Panx2), a membrane channel protein showing tumor-suppressing properties in cancer cells. Using a combination of subcellular fractionation, particle tracking in live-cell, and immunogold electron microscopy, we show that Panx2 localizes at ER-mitochondria contact sites in mammalian cells and sensitizes cells to apoptotic stimuli.

## 1. Introduction

Mitochondria are physically and functionally associated with the endoplasmic reticulum (ER) at membrane contact sites (MCSs) referred to as mitochondria-associated ER membranes (MAMs). ER-mitochondria MCSs are involved in lipid metabolism, mitochondrial calcium homeostasis, cellular respiration, mtDNA replication, mitochondrial division, autophagosome formation, and initiation of apoptosis [1,2,3,4,5,6,7,8]. MCSs constitute important signaling hubs and alterations in ER-mitochondria contacts have been associated with tumor progression and metastasis (reviewed in [9,10,11,12,13,14]). Recent studies have revealed that ER-mitochondria contacts can dynamically recruit oncogenes and tumor suppressors that modulate the bioenergetic demand, metabolism, and survival mechanisms of cancer cells. For example, Giorgi and co-workers recently demonstrated that in response to stresses, the tumor suppressor p53 accumulates at ER-mitochondria contacts where it interacts with the sarco-endoplasmic reticulum Ca^2+^ ATPase (SERCA) pump to promote mitochondrial calcium overload leading to apoptosis [15,16]. Similarly, Betz and co-workers have shown that upon stimulation by growth factors, mTORC2 localizes at ER-mitochondria contacts where it phosphorylates and activates Akt [17]. In turn, this mTORC2-Akt signaling regulates MAM integrity, ER-mitochondria Ca^2+^ flux, and energy metabolism by phosphorylating downstream targets [17].

The morphological features of ER-mitochondria MCSs are conserved across eukaryotes including plants [18,19], fungi [20,21,22], and mammalian cells [2,6,23]. However, despite this similarity, ER-mitochondria contacts are molecularly diverse. In yeast, ER-mitochondria contacts are mediated by the ER-mitochondria encounter structure (ERMES) complex [20]. However, this complex is not evolutionary conserved and is absent in metazoans. In mammalian cells, several proteins have been implicated as constituents of ER-mitochondria contacts such as the phosphofurin acidic cluster sorting protein 2 (PACS-2), the sigma-1 receptor (sigma-1R), mitofusin-2 (MFN2), the voltage dependent anion channel 1 (VDAC1), and the inositol 1,4,5-trisphosphate-gated receptor (IP3R) [24,25,26,27]. However, the precise molecular composition, diversity, and contribution of mammalian ER-mitochondria MCSs in cancer biology remains largely undefined.

Interestingly, we recently showed that Panx2, a gap junction with tumor-suppressing properties [28], forms puncta that localize to unidentified cytoplasmic compartments in mammalian cells [29]. Pannexins constitute a small family of membrane channel proteins (Panx1, Panx2, and Panx3) homologous to the innexins, which are invertebrate gap junction proteins [30]. However, unlike innexins and connexins, pannexins rarely participate in direct cell-cell coupling and instead primarily form non-junctional membrane channels [31]. Panx1 and Panx3 channels have been shown to localize partially to the ER where they form calcium leak channels [32,33] thereby raising the possibility that Panx2 also localizes to ER membranes. In this study, we employed a combination of subcellular fractionation, particle tracking in live-cell, and immunogold electron microscopy and discovered that the gap junction protein Panx2 is an ER-protein that localizes at ER-mitochondria contact sites. Functional data indicate that Panx2 over-expression sensitizes cancer cells to apoptotic signals. Thus, these findings identify Panx2 as a novel channel at the ER-mitochondria interface and a new player in the regulation of apoptosis.

## 2. Results

### 2.1. Panx2 forms Discrete Puncta on ER Membranes

**Figure 1:**
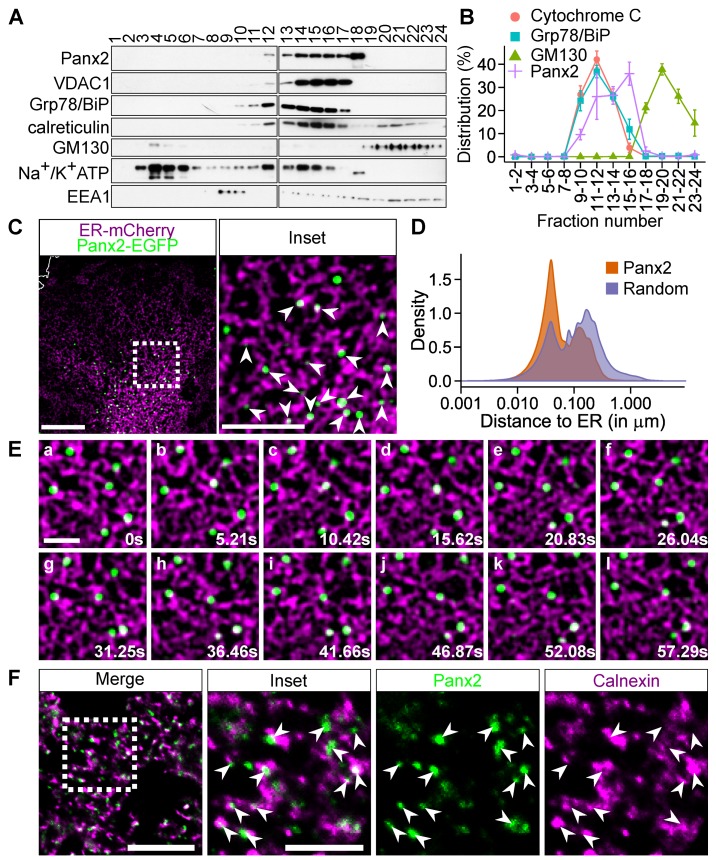
Pannexin 2 (Panx2) clusters in endoplasmic reticulum (ER) microdomains. (**A**) Panx2 co-fractions with ER and mitochondrial markers. Post-nuclear supernatant from C6 Panx2-enhanced green fluorescent protein (EGFP) cells was fractionated by ultracentrifugation and twenty-four fractions were collected and immunoprobed for various organelle markers. A substantial amount of Panx2 co-fractionated with mitochondrial (voltage dependent anion channel 1 (VDAC1) and ER (Grp78/BiP, calreticulin) markers. (**B**) Three additional fractionations were performed but fractions were paired to allow the probing of all fractions on a single membrane for quantification. Panx2 distribution positively correlated (Pearson’s correlation) with the mitochondrial marker cytochrome C (r = 0.633, *p* = 0.0273) and the ER protein Grp78/BiP (r = 0.76, *p* = 0.0041) but not with the Golgi marker GM130 (r = −0.407, *p* = 0.1892). (**C**) C6 Panx2-EGFP cells were transfected with an ER-targeted mCherry construct and trajectories of Panx2 puncta were tracked in living cells. A large majority of Panx2 puncta were located on the ER network as indicated by the white arrowheads (inset). Scale bars: 10 µm and 5 µm (inset). (**D**) The probability distribution of Panx2 puncta (orange curve) was compared to the probability distribution calculated after randomizing the trajectories of Panx2 puncta within the cytoplasm (purple curve). The distributions, calculated from 13 cells, show that a larger proportion of Panx2 puncta was localized on the ER network prior to randomization. (**E**) Frame sequence showing that Panx2-ER association was stable for over 55 s despite the high mobility of the ER network during that period. Scale bar, 2 µm. (**F**) Mouse brain sections were stained for Panx2 and the ER marker calnexin. Panx2 formed discrete puncta that primarily clustered in ER microdomains as indicated by the white arrowheads (inset). Scale bars, 10 µm (inset 5 µm).

We and others have previously reported that Panx2 localization is unique among plasma membrane junction proteins as it distributes as membrane-bound puncta within unidentified cytoplasmic compartments [29,34,35,36,37,38]. To determine the identity of these Panx2 structures, we analyzed their distribution in postnuclear supernatants fractionated by density gradient ultracentrifugation. While a substantial portion of Panx2 was found in fraction # 18 which was not enriched for other organelle markers (Figure 1A), protein profiling of Panx2 co-fractionated with bonafide ER and mitochondrial proteins (Figure 1A,B). The composition of fraction # 18 remains elusive, but might constitute a population of microsomes specifically enriched in Panx2. Unlike its paralogs Panx1 and Panx3, the *N*-glycosylated high mannose residues of Panx2 are not processed into complex oligosaccharides by Golgi-specific enzymes [35], thereby suggesting that Panx2 does not transit through the Golgi but primarily localizes to the ER, consistent with our fractionation data.

To further test this observation, we used time lapse confocal microscopy to examine the localization of Panx2 relative to the ER in C6 cells stably expressing Panx2-enhanced green fluorescent protein (EGFP) [28] and transfected with ER-targeted mCherry (ER-mCherry). We tracked the trajectories of Panx2 puncta and calculated the probability distribution of Panx2 in relation to the surrounding ER network over time. Our analysis indicates that Panx2 puncta are persistently localized with the ER (Figure 1C,D). To determine whether this observation was a stochastic event attributable to the dense ER network, we compared the probability distribution before and after randomizing the trajectories of Panx2 puncta within the cytoplasm. A much larger proportion of Panx2 puncta was associated with the ER prior to randomization thereby confirming that Panx2 puncta are distributed on ER membranes in a non-random manner (Figure 1D). The non-stochastic nature of the Panx2-ER association was further tested by comparing the cumulative distribution of Panx2 puncta before and after randomization. A larger proportion of Panx2 puncta distributed within 100 nm of an ER tubule prior to randomization, consistent with an ER-localization of Panx2 foci (70.82 ± 5.60% prior to randomization vs 46.95 ± 6.75% after randomization, *p*-value = 1.005 $\times 10^{-9}$, paired *t*-test, n = 13). In addition, we observed that Panx2 puncta association with ER membranes is also stable despite the high dynamism of the ER network (Figure 1E). Examination of endogenous Panx2 localization in vivo in brain sections by indirect immunofluorescence revealed that endogenous Panx2 was present as clustered puncta associated with ER membranes (Figure 1F, see also Supplementary Figures S1 and S2). Thus, together our data indicate that Panx2 forms focal structures associated with the ER in mammalian cells.

### 2.2. Panx2 Puncta Localize at ER-Mitochondria Contact Sites

**Figure 2:**
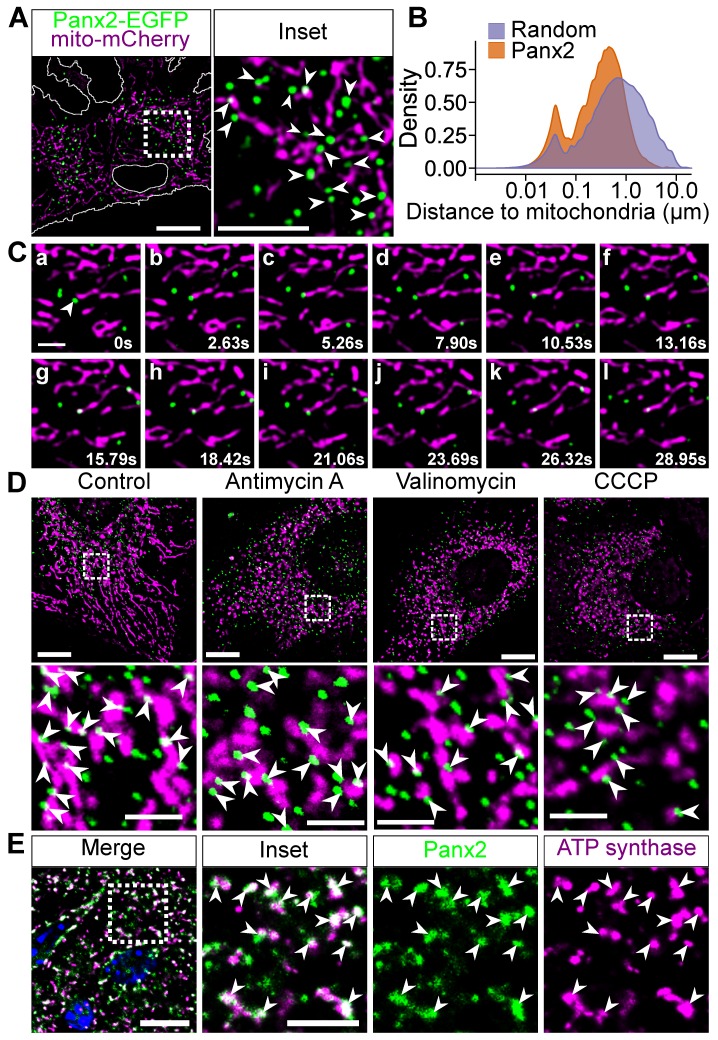
Panx2 puncta associate with mitochondria. (**A**) The dynamic of Panx2 puncta was analyzed in C6 Panx2-EGFP cells transfected with mitochondria-targeted mCherry. Many Panx2 puncta were closely associated with the mitochondrial network as indicated by the white arrowheads (inset). Scale bar, 10 µm. (**B**) The probability distribution of Panx2 puncta was calculated before and after randomizing the trajectories of Panx2 puncta within the cytoplasm (orange and purple curves respectively). The distributions, computed from 17 cells, show that Panx2 puncta were not randomly distributed within the cytoplasm; more Panx2 foci localized in the proximity of or in direct apposition with mitochondria that can be explained by chance. (**C**) Frame sequence showing that Panx2 association with mitochondria is stable even though the mitochondrial network is dynamic. A Panx2 puncta approaching a mitochondrion in frame a (white arrowhead) established contact with the organelle in frames b and c. The association was briefly lost (frame d) but stably re-established for over 18 s from frame e onward. Scale bar, 2 µm. (**D**) C6 Panx2-EGFP cells were stained with MitoTracker CMXRos and either untreated (control) or treated with antimycin A (40 µM) or the mitochondrial uncouplers valinomycin and carbonyl cyanide m-chlorophenyl hydrazone (CCCP) (100 nM and 10 µM, respectively) for 2 h prior fixing. Several Panx2 puncta (green) remained associated with mitochondria (magenta) following the fragmentation of the mitochondrial network upon treatment with antimycin A, valinomycin, and CCCP as indicated by the arrowheads. Scale bars: 10 µm (top panels) and 2.5 µm (insets). (**E**) Mouse brain sections were stained for Panx2 and ATP synthase α, a subunit of mitochondrial complex V. A large proportion of Panx2 staining overlapped with brain mitochondria as indicated by the white arrowheads (inset). Scale bars, 10 µm (inset 5 µm).

Interestingly, Panx2 also co-fractioned with mitochondrial markers (Figure 1A,B), thereby suggesting that Panx2 puncta may interact, at least transiently, with mitochondria. To test this hypothesis, we transfected Panx2-EGFP C6 cells with mitochondrial matrix-targeted mCherry (mito-mCherry) and analyzed the distribution of Panx2 foci relative to mitochondria using time lapse confocal microscopy. Panx2 puncta were shown to be spatially linked with mitochondria (Figure 2A). The probability distribution analysis of Panx2 puncta in relation to the mitochondrial network confirmed the non-stochasticity of Panx2 association with mitochondria (Figure 2B). A larger proportion of Panx2 puncta was associated with mitochondria prior to randomizing the trajectories of Panx2 puncta within the cytoplasm (Figure 2B). The association of Panx2 with mitochondria was further confirmed by calculating the cumulative distribution function before and after randomization. Panx2 puncta had a significantly higher probability to be localized within 100 nm of a mitochondrion before randomizing their trajectories thereby further substantiating the non-stochasticity of the association (21.74 ± 8.08% prior to randomization vs. 13.44 ± 3.58% after randomization, *p*-value = 0.0008251, paired *t*-test, n = 17). The association between Panx2 puncta and mitochondria was also stable despite the mobility of the mitochondrial network (Figure 2C). Furthermore, some Panx2 foci remain associated with mitochondria following mitochondria fragmentation induced by the respiratory complex inhibitor antimycin A or the ionophores valinomycin or carbonyl cyanide m-chlorophenyl hydrazone (CCCP) (Figure 2D) thereby reinforcing the idea of a physical interaction between Panx2 focal structures and mitochondria. We also confirmed that endogenous Panx2 also distributed as puncta that were closely associated, and often overlapping, with mitochondria in vivo (Figure 2E). Thus, our data indicate that Panx2 focal structures on ER membranes associate with mitochondria.

**Figure 3:**
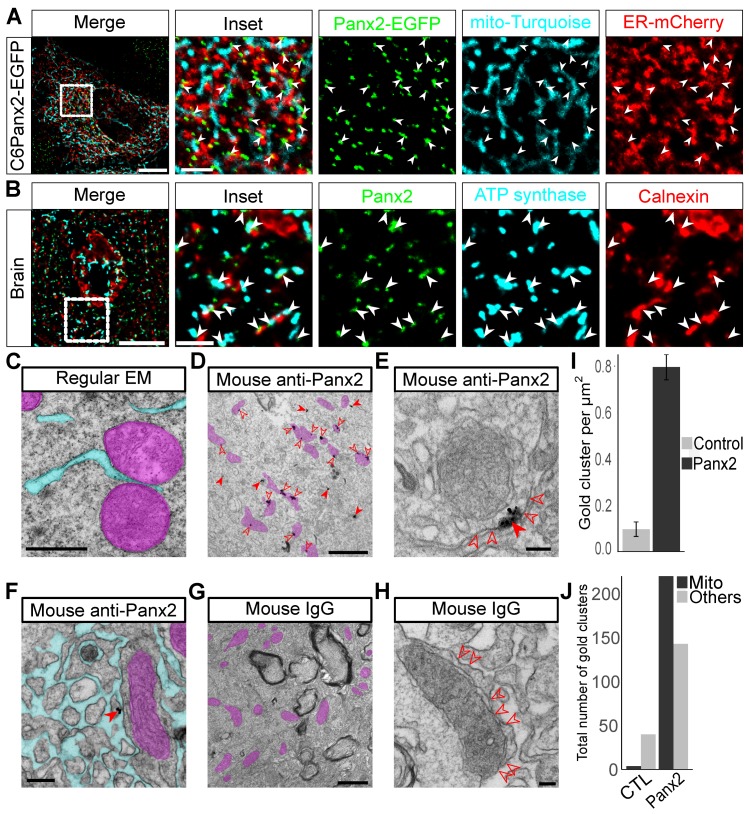
Panx2 localizes at ER-mitochondria contact sites in vivo. (**A**) C6 Panx2-EGFP cells were co-transfected with mito-Turquoise and ER-mCherry. Many Panx2 puncta simultaneously contact ER and mitochondrial networks as indicated by the white arrowheads. Scale bars: 10 µm (inset 2.5 µm). (**B**) Mouse cortical brain sections were co-stained for Panx2, the ER marker calnexin and the mitochondrial protein ATP synthase. Panx2 is found on ER microdomains that are closely associated with mitochondria in vivo as indicated by the white arrowheads. Scale bars: 10 µm (inset 2.5 µm). (**C**) Transmission electron micrograph of a mouse cortical brain showing that the gap between ER and mitochondria is often imperceptible at ER-mitochondria contact site. (**D**) Representative image of a mouse brain cortex showing the localization of Panx2 by immunogold. Most gold clusters are found in close association with mitochondria (open arrowheads). Gold clusters not located near mitochondria (solid arrowheads) are located on ER membranes as seen in F. (**E**) Representative example of an immunogold staining from mouse brain cortex showing the presence of Panx2 at ER-mitochondria contact sites (solid arrowhead). Gold particles are in the narrow gap which defines the cytoplasmic region between ER (open arrowheads) and mitochondrial membranes. (**F**) Representative image of a mouse brain cortex showing the localization of Panx2 (solid arrowhead) on ER membranes by immunogold staining. The gold particles are in the cytoplasm and not inside the ER lumen which is consistent with the localization of Panx2 C-terminal tail. (**G**,**H**) Representative electron micrographs from mouse brain cortex illustrating the absence of gold particles when the anti-Panx2 primary antibody was replaced by an antibody from a non-immunized mouse. The localization of the ER membranes at Mitochondria-associated ER membranes (MAMs) is indicated by open arrowheads. The ER lumen was pseudocolored in cyan (**C**,**F**) and mitochondria in purple (**C**,**D**,**F**,**G**). Scale bars: 500 nm (**C**), 1 µm (**D**,**G**), 100 nm (**E**,**H**) and 200 nm (**F**). (**I**) Brain sections immunoprobed for Panx2 had a much higher density of gold clusters than control sections immunoprobed with an antibody from a non-immunized mouse (respectively 0.796 ± 0.055 vs. 0.097 ± 0.031 cluster per µm^2^). (**J**) In cortical brain sections immunoprobed for Panx2, 220 out of 363 gold clusters (60.61%) were directly associated with mitochondria. In contrast, only 4 gold clusters were found associated with mitochondria in cortical sections from control brains. Two different brains and a total of forty-one images per group were used for quantification in I and J. Both brains were used in each group.

Since our data indicate that Panx2 is dually localized to mitochondria and the ER, we considered whether Panx2 localizes to ER-mitochondria MCSs [39,40,41]. The localization of MCS-associated proteins are often distributed non-uniformly and instead concentrate at points of close physical proximity between contact site membranes thereby adopting a punctate or patchy distribution [20,22,42], similar to what we observed for Panx2. Thus, we tested whether Panx2 was localized at ER-mitochondria MCSs by imaging Panx2-EGFP C6 cells co-transfected with the mitochondrial matrix-targeted Turquoise (mito-turquoise) and ER-mCherry. We observed that Panx2-EGFP puncta often associate with both ER and mitochondrial markers (Figure 3A). In addition, we co-labeled Panx2, ER, and mitochondria in mouse brain sections and observed that puncta formed by endogenous Panx2 were localized at regions where ER and mitochondria membranes were in close proximity (Figure 3B). The distribution of endogenous Panx2 clusters at ER-mitochondria contact sites was also observed using anti-Panx2 antibodies raised in different species (Supplementary Figure S1). Thus, together our data suggest that Panx2 localizes at ER-mitochondria contact sites.

To further test whether Panx2 is localized to ER-mitochondria MCSs, we examined Panx2 endogenous localization at the ultrastructural level by performing quantitative immunogold electron microscopy on mouse cortical brain sections. We observed that a majority (60.61%) of gold particles was directly associated with mitochondria (Figure 3D–J). In most instances, gold particles were observed directly positioned on the outer and inner mitochondrial membranes or in the mitochondrial matrix (Figure 3D). However, this is likely explained by the physical dimension of the antibody complex and colloidal gold particle which was subsequently enhanced by silver staining [43]. As a result, silver-enhanced gold clusters can be located tens of nanometers away from the epitope. However, when ER and mitochondrial membranes could be successfully resolved, gold particles were shown to localize in the narrow cytoplasmic cleft separating both organelles thereby confirming that Panx2 puncta localize on ER membranes at MAM sites and not on mitochondrial membranes (Figure 3E). Moreover, gold particles not juxtaposed to mitochondria were shown to be located on ER membranes (Figure 3F) which further confirms that Panx2 forms discrete puncta on ER membranes and not on mitochondrial membranes. We did not identify gold clusters in association with other organelles beside ER and mitochondria.

**Figure 4:**
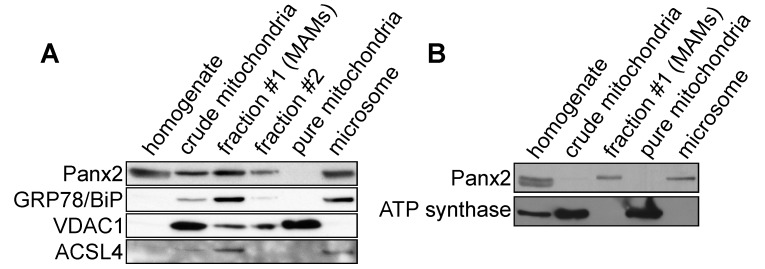
Panx2 is enriched in purified MAMs but is absent from pure mitochondria. (**A**) MAMs and pure mitochondria were isolated from mouse liver and immunoprobed with different markers. Two distinct bands, labeled fraction #1 (MAMs) and fraction #2, were isolated above the mitochondrial fraction by ultracentrifugation on a Percoll gradient. Panx2 is found in the crude mitochondrial and MAM fractions but not the pure mitochondrial fraction. Purified MAMs were also enriched in Grp78/BiP and ACSL4. (**B**) Isolated MAMs are enriched in Panx2 but are devoid of inner mitochondrial membrane contaminant as shown by the absence of ATP synthase.

MAMs have been enriched from crude mitochondrial preparations by subcellular fractionation [44,45]. Using this protocol, we observed that Panx2 from mouse liver extracts was co-enriched with the MAM markers Grp78/BiP, an ER chaperone, and the long-chain acyl-CoA synthetase 4 (ACSL4) protein, an enzyme involved in lipid biosynthesis (Figure 4A) [25,45]. Although we observed Panx2 in crude mitochondrial fractions it was not detectable in fractions further enriched for mitochondria (Figure 4). Thus, these biochemical fractionation data are consistent with Panx2 being an ER protein localized to ER-mitochondria MCSs. Interestingly, the outer mitochondrial membrane protein VDAC1 was detected in MAMs (Figure 4A) while the inner mitochondrial membrane protein ATP synthase was absent from the isolated MAMs (Figure 4B). This suggests that ER and mitochondrial membranes might remain tethered during the isolation procedure and strips some of the outer membrane from mitochondria. We also observed two Panx2 bands in the homogenate but not the MAM fractions (Figure 4B) thereby suggesting that post-translational modifications might influence Panx2 localization at MAMs. As Panx2 is glycosylated to a high-mannose form [35], it is possible that only the mature high-mannose type localizes at MAMs.

### 2.3. Panx2 Sensitizes Cells to Apoptosis

**Figure 5:**
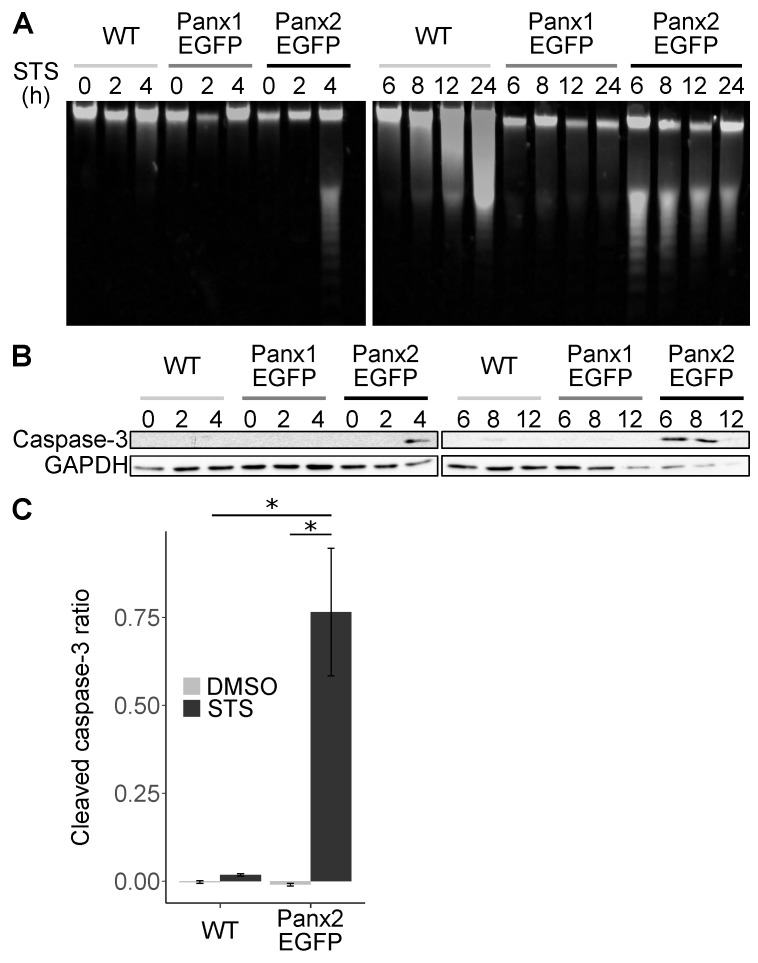
Panx2 sensitizes glioma cells to apoptosis. (**A**) C6 wildtype (WT), C6-Panx1-EGFP and C6 Panx2-EGFP glioma cells were treated for 2 h to 24 h with 1 µM staurosporine (STS) prior to assessing apoptosis by DNA gel electrophoresis. C6 cells expressing Panx2 underwent apoptosis as soon as 4 h after STS application as showed by evidence of DNA fragmentation. In contrast, C6WT and C6-Panx1-EGFP cells did not show appreciable sign of DNA fragmentation within that period. (**B**) C6 WT, C6-Panx1-EGFP and C6 Panx2-EGFP cells were treated with 1 µM STS for 2–12 h prior to immunoblotting for cleaved caspase-3. C6 Panx2-EGFP, but not C6WT and C6-Panx1EGFP, showed strong activation of caspase-3 starting 4 h after STS treatment. (**C**) The normalized intensity of cleaved caspase-3 staining following 1 µM STS treatment for 6 h was analyzed by a two-way mixed factorial ANOVA, with cell type and treatment as independent variables (n = 3). A logarithmic transformation was used to ensure normal distribution of the residuals, homoscedasticity was confirmed using the Levene’s test, and statistical significance was further examined using a post hoc Tukey test. A significant effect, indicated by *, was found for the cell type F_0.05_(1,8) = 12.07 *p*-value < 0.0084, STS treatment F_0.05_(1,8) = 67.11 *p*-value < 3.7 × 10^−5^, and the interaction F_0.05_(1,8) = 32.42 *p*-value < 0.0005. This indicates that STS treatment induced a significant increase in caspase-3 activation in C6 Panx2-EGFP but not C6WT cells.

ER-mitochondria MCSs have been shown to regulate Ca^2+^ exchange between ER and mitochondria [46,47,48] and are integral components of intrinsic apoptosis upon mitochondria Ca^2+^ overload [8,23,49,50,51]. Thus, we examined whether Panx2 played a functional role in the regulation of apoptosis. Specifically, we examined the sensitivity of cells to staurosporine (STS)-induced apoptosis in cells over-expressing Panx2 (Figure 5). Cells over-expressing Panx2 showed signs of accelerated apoptosis after STS application relative to control cells. DNA fragmentation and activation of caspase-3 was observed at 4h as compared to 8h for control cells (Figure 5A–C). In addition, C6 cells over-expressing the paralog Panx1, were comparable in their apoptotic response to control C6 cells, which do not express detectable amount of endogenous Panx2 [28,29] (Figure 5A–C). We tested different cell lines (A549, U2OS, 293T, MEF, HepG2, NRK, NIH-3T3) but have not identified a cell line unequivocally and consistently expressing endogenous Panx2 in culture. Thus, we could not perform gene silencing experiments to examine whether Panx2 was required for apoptosis. Our results however suggest that Panx2 can promote intrinsic apoptosis.

## 3. Discussion

We and others had previously reported that Panx2 does not localize at the plasma membrane like other gap junction proteins but primarily localizes in membrane–bound compartments within the cytoplasm [28,29,34,37,38]. Here, we characterized the nature of these membrane-bound compartments by subcellular fractionation, analyzing Panx2 dynamics in living cells, and performing immunogold electron microscopy on brain sections. We demonstrated that Panx2 is an ER protein that does not randomly distribute within the ER network but forms focal structures that localize at ER-mitochondria contact sites. Our results on C6 cells over-expressing Panx2 indicate that about 20% of Panx2-EGFP foci localize to mitochondria. Interestingly, our immunogold data indicate that under physiological conditions and endogenous expression, the majority of Panx2 foci (~60%) are directly associated with mitochondria thereby suggesting that over-expressing Panx2 might saturates Panx2-containing MAM sites and lead to the accumulation of Panx2 on ER membranes that are not associated with mitochondria.

While our study identifies Panx2 as a novel component of the mammalian ER-mitochondria MCSs, other groups have suggested that ectopic Panx2 localizes within endolysosomal compartments [34,37,52]. However, the glycosylation pattern of Panx2 is inconsistent with its trafficking to endosomal compartments. While all pannexin paralogs are glycosylated into high mannose species within the ER, only Panx1 and Panx3 proteins are processed into complex N-glycoproteins [35,53,54,55] thereby suggesting that Panx2 does not transit through the Golgi and the secretory pathway. We did not find evidence of endolysosomal localization (Supplementary Figure S3) but cannot exclude the possibility that a small fraction of Panx2 puncta localizes in the vicinity of endocytic organelles under certain conditions. ER tubules have been shown to form tight and stable contact sites with endosomes [56,57,58] and ER proteins can dually localize to ER-mitochondria and ER-vacuole contact sites in yeast [22]. However, our immunogold data show that the majority of endogenous Panx2 is clustered on ER membranes directly associated with mitochondria and is not distributed in the immediate proximity of other organelles. Therefore, our data do not support the localization of Panx2 at other ER contact sites under physiological conditions.

We previously showed that Panx2 expression reduces in vitro and in vivo oncogenicity properties [28]. Over–expressing Panx2 protein in C6 rat glioma cells reduced cell proliferation and saturation density, suppressed anchorage–independent growth and decreased in vivo tumor growth [28]. Panx2 localization at ER-mitochondria contact sites likely contributes to its tumor-suppressing properties. Owing to its channel properties, Panx2 might facilitate the exchange of ions and small molecules between ER and mitochondria at MCSs. The close apposition of ER and mitochondrial membranes at MAMs allows the propagation of Ca^2+^ signals from the ER to mitochondria [23,48]. The IP3Rs and ryanodine receptors (RyRs) are two major ER Ca^2+^ channels which have been shown to influence mitochondrial Ca^2+^ uptake [59,60]. However, silencing IP3Rs in cells that do not express RyRs only partially reduced mitochondrial Ca^2+^ uptake [60], thereby indicating that additional channels must also regulate Ca^2+^ exchange at ER-mitochondria contact sites. Interestingly, Panx2 paralogs form Ca^2+^-permeable ER channels in over-expressing systems [32,33], thereby suggesting that Panx2 channels might be involved in Ca^2+^ exchange between the ER and mitochondria. In this context, we speculate that Panx2 can regulate apoptosis by triggering ER Ca^2+^ release events that compromise mitochondrial Ca^2+^ homeostasis and initiates apoptotic programs [23,61]. Panx2 could also regulate apoptotic programs independently of its channel activity. Panx2 is characterized by a long (~360 amino acids) and intrinsically disordered C-terminal tail facing the cytoplasm. Thus, Panx2 could directly or indirectly interact with mitochondrial proteins and promote the tethering of ER to mitochondrial membranes. The association of the ER with mitochondria is dynamically regulated and can influence the cellular response to apoptotic signals. Molecular tethers that couple ER and mitochondria display some diversity in length which influences the distance between both organelles [23]. The tightening of ER-mitochondria contacts increases the functional association between both organelles and increases mitochondrial Ca^2+^ uptake [23]. Under cellular stresses, excessive tightening of ER-mitochondria contacts can dysregulate mitochondrial Ca^2+^ homeostasis and trigger cellular apoptosis [23]. Interestingly, another group recently found that silencing Panx2 expression sensitizes pancreatic -cells to cytokine-induced apoptosis in vitro [62]. In this study, Berchtold and coworkers showed that Panx2 mRNA expression was downregulated by interleukin-1 and interferon- and that further silencing Panx2 mRNA expression by siRNA delivery aggravated cytokine-induced apoptosis in rodent -cells [62]. This effect was mediated by an increase in cytokine-induced expression of inducible nitric oxide synthase upon Panx2 silencing [62]. Interestingly, Panx2 silencing increased cytokine-mediated cell death but did not sensitize cells to thapsigargin-induced ER stress [62]. However, while these results suggest that the anti-apoptotic properties of Panx2 might be independent of its localization at MAMs they should also be interpreted with caution since we have previously shown that Panx2 transcriptional activity is a poor predictor of Panx2 protein abundance and does not correlate with Panx2 protein levels [29]. Therefore, without directly measuring Panx2 protein levels, it is difficult to assess the physiological impact of Panx2 mRNA silencing.

## 4. Materials and Methods

### 4.1. Animal Care

All experiments were performed in accordance with the guidelines established by the Canadian Council on Animal Care and were approved by the University of British Columbia Animal Care Committee (The approval code is A15-0228).

### 4.2. Antibodies

The anti-Panx2 mouse monoclonal antibody (cat# 75-212, clone N121A/1) was from UC Davis/NIH NeuroMab Facility (Davis, CA, USA) and was used at 2 µg/mL for immunofluorescence (IF), 5 µg/mL for immunogold and 4 µg/mL for Western blot (WB). The chicken anti-Panx2 antibody (cat# ANT0040, IF: 1/1000) was from Diatheva (Fano, Italy). The purified immunoglobulin from non-immunized mouse was obtained from Jackson Immunoresearch (cat# 015-000-003; West Grove, PA, USA) and was used at the same concentration as the anti-Panx2 antibody. Mouse monoclonal antibodies against EEA1 (cat# 610456, IF: 1/250, WB: 1/5000), p47a (cat# 610890, IF: 1/250), Rab4 (cat# 610888, IF: 1/250) and GM130 (cat# 610822, IF: 1/250, WB: 1/1000) were all from BD Biosciences (Franklin Lakes, NJ, USA). The monoclonal antibody against Stim1 (cat# 49-119, IF: 1/100) was from ProSci (Poway, CA, USA). The monoclonal antibodies against ATP synthase subunit (cat# A21350, IF: 1/400, WB: 1/1000) was from Thermo Fisher Scientific (Rockford, IL, USA). The anti-Na^+^/K^+^ ATPase antibody (cat# ab7671, WB: 1/5000) was from Abcam (Cambridge, United Kingdom). The anti-cleaved caspase-3 antibody was from Cell Signaling Technology (Danvers, MA, USA) (cat# 9661, WB: 1/1000). The anti-SKL antibody was kindly provided by Dr Richard Rachubinski from the University of Alberta and was used at 1:250 for immunofluorescence. The anti-sec16 antibody was kindly provided by Dr Ivan Robert Nabi from the University of British Columbia. The anti-GRP78/BiP antibody (cat# G8918, WB: 1/3000), the anti-calnexin (cat# C4731, IF: 1/200, WB: 1/2000), and HRPO-conjugated goat secondary antibodies were obtained from Sigma (St. Louis, MO, USA). AlexaFluor488-conjugated goat anti-mouse IgG2a (cat# A21131), AlexaFluor 594-conjugated goat anti-mouse IgG3 (cat# A21155) and other AlexaFluor- conjugated secondary antibodies were from Thermo Fisher Scientific and were used at 1/400. CF594-conjugated goat anti-mouse IgG2b secondary antibody (cat# 20269, IF: 1/400) was from Biotium (Hayward, CA, USA). The nanogold anti-mouse Fab’ (cat# 2002) secondary antibody was from Nanoprobes (Yaphank, NY, USA) and was used at 1/100.

### 4.3. Plasmids Construction

An expression vector encoding a mitochondrial matrix-targeted mCherry (mito-mCherry) was generated by modifying a DsRed-mito plasmid [63] by restriction free cloning. The mCherry sequence was initially subcloned from a mCherry-miniSOG-N1 plasmid kindly provided by Dr Roger Tsien from the University of California, San Diego using the following forward and reverse primers respectively: 5′-AGTCCAGAGTCAAGTACAGCTG GGATCCATGGTGAGCAAGGGCGAG-3′ and 5′-GGCCCTCTAGAGCGGCCGCTTACTTGTACAGCTCGTCCATGC-3′. The reaction product was purified using the QIAquick gel extraction kit (Qiagen, Venlo, Limburg, Netherlands) and used in a secondary PCR reaction with the DsRed-mito plasmid as template, thereby replacing the DsRed sequence by the mCherry gene. The mito-Turqoise construct was designed similarly. Forward 5′-CCAGAGTCAAGTACAGCTGGGATCCATGGTGAGCAAGGGCGAG-3′ and reverse 5′-TAGGGCCCTCTAGAGCGGCCGCTTACTTGTACAGCTCGTCCATGC-3′ primers were used to amplify the Turquoise2 sequence from the pmTurquoise2-Golgi plasmid obtained from Addgene (cat# 36205; Cambridge, MA, USA). The amplification product was subsequently used in a second PCR reaction with the mito-mCherry plasmid as template thereby replacing the mCherry gene with the Turquoise2 sequence. The ER-targeted mCherry expression vector was also engineered by restriction free cloning. A set of primers were used to amplify mCherry (forward: 5′-CTGGGCGCCGCCGCCGACATGGTGAGCAAGGGCGAG-3′, reverse: 5′-GATGGATATCTGCAGAATTCTTACAGCTCGTCCTTCTTGTACA GCTCGTCCATGCC-3′) and the reaction product was used in a second PCR with the pcDNA3-D1ER plasmid from Dr Roger Tsien as template. These steps essentially swapped the cameleon calcium sensor sequence for the mCherry gene while keeping the calreticulin ER-targeting and KDEL ER-retention signals. All PCR reactions were carried out using Phusion high fidelity DNA polymerase (Thermo Fisher Scientific).

### 4.4. Cell Culture

Rat C6 glioma, C6 Panx1-EGFP, and C6 Panx2-EGFP were grown in Dulbecco’s Modified Eagle Medium (DMEM) (cat# D6429, Sigma) supplemented with 10% fetal bovine serum (FBS) and cultured at 37 ${}^{\circ }$C and 5% CO_2_.

### 4.5. Live-Cell Imaging and Panx2 Trajectory Analysis

The day prior to imaging, C6 Panx2-EGFP cells plated on 35 mm (No 1.5) glass bottom dishes (MakTek Corporation, Ashland, MA, USA) were transfected with ER-mCherry, mito-mCherry or mito-Turquoise constructs using Lipofectamine 2000 or Lipofectamine 3000 (Thermo Fisher Scientific) according to the manufacturer’s instructions. A few minutes before imaging, cells were labeled for 5 min with CellMask Deep Red plasma membrane stain at 2.5 µg/mL to 5 µg/mL (Thermo Fisher Scientific). The green fluorescent protein (GFP) and mCherry channels from transfected cells were acquired simultaneously using a Leica TCS SP5 confocal microscope with a 63× objective (1.4 NA) (Leica, Mannheim, Germany). The far-red channel from the same optical section was also captured to record the plasma membrane and delimit the cytoplasm boundaries. Live-cell imaging was performed in complete media supplemented with 25 mM HEPES at 37 ${}^{\circ }$C. Time-lapse images were processed with an iterative Tikhonov-Miller deconvolution algorithm [64] and the movement of Panx2 puncta was analyzed using the particle tracker plugin from the MosaicSuite for ImageJ [65]. In house software written in R was used to randomize the distribution of Panx2 trajectories within the cytoplasm and to compute the minimal distance between the centroid of individual Panx2 puncta and mCherry-labeled organelles. The R codes were formatted into a R package which can be installed from GitHub (www.github.com/MaxLev/CloseEnough) and a R vignette is also available CloseEnough.

### 4.6. Immunofluorescence and Co-Localization Analysis

Adult mice were transcardially perfused with 5 mL of heparinized saline followed by 30 mL of 4% paraformaldehyde and 0.1% glutaraldehyde in 0.1 M phosphate buffer (PB). Brains were sliced in 2 mm to 3 mm thick sections, postfixed for 4 h in 4% paraformaldehyde at 4 ${}^{\circ }$C, equilibrated overnight in 30% sucrose in 20 mM phosphate buffer saline (PBS), embedded in Tissue-Tek O.C.T. (Sakura Finetek, Torrance, CA, USA), frozen and cryosectioned at 10 µm. Tissue sections were washed in 20 mM PBS and antigen retrieval was performed by incubation in 10 mM sodium citrate (pH 8.5) pre-warmed at 80 ${}^{\circ }$C for 2.5 min. Samples were then washed with PBS, treated with 0.1% sodium borohydride in PBS for 30 min and further washed with PBS. Samples were blocked for 1 h at room temperature with 5% goat serum or 5% bovine serum albumin (BSA) and incubated overnight at 4 ${}^{\circ }$C in primary antibody diluted in blocking solution. The samples were then washed in PBS and incubated for 1 h at room temperature with secondary antibodies diluted in blocking solution, followed by mounting in ProLong Gold or ProLong Diamond antifade reagent with 4′,6-diamidino-2phenylindole (DAPI) (Thermo Fisher Scientific). Cells grown on coverslips were simply fixed with 4% paraformaldehyde in 0.1 M PB for 20 min at room temperature, permeabilized with 0.2% Triton X-100 in 20 mM PBS prior blocking and immunolabeled as described above. The labeling duration was shortened to 1h at room temperature for cells grown on coverslips. Imaging was performed on a Leica TCS SP5 confocal microscope with a 63× objective (1.4 NA). Images were further processed with an iterative Lucy-Richardson deconvolution algorithm [64]. All images were displayed as individual optical sections taken from a z-stack and not as maximal projection. The Mander’s coefficients were calculated using the JACoP ImageJ plugin as previously described [66].

### 4.7. Pre-Embedding Immunoelectron Microscopy

For pre-embedding immunogold on mouse brain cortex, sections were processed as indicated above for conventional immunofluorescence but sections were stored at −20 ${}^{\circ }$C in 30% glycerol, 30% ethylene glycol in 20mM PB following cryosectioning. Pre-embedding immunogold staining was performed as previously described [67]. Briefly, following the antigen retrieval and sodium borohydride treatment, sections were blocked for 1 h in 5% BSA in 20 mM PBS, incubated with the primary antibody diluted in blocking buffer for 1 h at room temperature and washed with PBS. Sections were then blocked with 5% BSA for an additional 30 min before incubating them with goat anti-mouse Fab’ fragments conjugated with 1.4 nm gold particles diluted in blocking buffer for 1 h at room temperature. Sections were then washed in 1% BSA in 20mM PBS followed by three additional washes in PBS. Sections were then postfixed in 2% glutaraldehyde for 10 min and washed thoroughly in distilled water. Gold particles were silver enhanced in the dark for 10 min using the HQ Silver kit (Nanoprobes) according to the manufacturer’s instructions. Sections were then washed thoroughly with distilled water and immersed in 5% sodium thiosulfate for 3 min. Sections were then postfixed in 1% osmium tetraoxide:1.5% potassium ferricyanide in 0.1 M sodium cacodylate buffer for 10 min and thoroughly washed with distilled water. Sections were then stained with 1% aqueous uranyl acetate for 5 min before dehydration through ascending concentrations of ethanol and embedding in EMBED 812 resin (Electron Microscopy Sciences, Hatfield, PA, USA). Thin sections were cut on a Leica EM UC7 ultramicrotome (Leica Microsystems), collected on 200 mesh copper grids (Electron Microscopy Sciences) and stained with uranyl acetate and lead citrate. Finally, 11.125 µm^2^ images were acquired on a Tecnai G2 Spirit electron microscope (FEI North America NanoPort) operated at 120 kV.

### 4.8. Subcellular Fractionation and MAM Isolation

The subcellular fractionation was performed as followed. Five 15cm confluent plates of C6 Panx2-EGFP cells were homogenized in 2 mL of 0.25M sucrose, 1mM ethylenediaminetetraacetic acid (EDTA) and 10 mM HEPES (pH 7.4) supplemented with protease inhibitors (Pierce, Rockford, IL, USA). The homogenate was centrifuged at 1500 g for 10 min and the supernatant was brought to a final concentration of 35% OptiPrep (Sigma Aldrich) using a 60% stock solution (60% OptiPrep, 0.25 M sucrose, 1 mM EDTA and 10 mM HEPES). Three milliters were loaded at the bottom of a tube and overlaid with 30%, 20%, 17.5%, 15%, 12.5%, 10%, 7.5%, 5%, and 2.5% of OptiPrep containing 0.25 M sucrose, 1 mM EDTA and 10mM HEPES (1ml each). The sample was then centrifuged at 200,000 g for 2.5 h and 0.5 mL fractions were collected and frozen until ready to use.

MAMs and pure mitochondria stripped from their associated membranes were isolated by subcellular fractionation as previously described [44]. Approximately 0.5 g of mouse liver was homogenized in 225 mM mannitol, 75 mM sucrose, 0.5% BSA, 0.5 mM ethylene glycol tetraacetic acid (EGTA) and 30 mM Tris-HCl pH 7.4 and a crude mitochondrial pellet was obtained by differential centrifugation. The crude mitochondrial pellet was resuspended in mitochondrial resuspension buffer (MRB) buffer (250 mM mannitol, 5 mM, HEPES pH 7.4 and 0.5 mM EGTA), overlayed on top of a Percoll medium (225 mM mannitol, 25 mM HEPES pH 7.4, 1 mM EGTA, 30% Percoll) and centrifuged at 95,000 g at 4 ${}^{\circ }$C for 30 min. Following centrifugation, the MAM fraction was identified as diffuse white bands located above the mitochondrial pellet. The diffuse white bands and mitochondrial pellet were collected separately, diluted ten times with MRB buffer, and centrifuged at 6300 g for 10 min. The supernatant of the mitochondrial pellet was discarded and the pellet containing pure mitochondria collected. The MAM supernatant was collected and centrifuged at 100,000 g to obtain a MAM-enriched pellet.

### 4.9. Western Blotting

Tissues or cells were homogenized in radioimmunoprecipitation assay (RIPA) buffer (150 mM NaCl, 25 mM Tris-HCl pH 8.0, 0.5% Sarkosyl, 1% IGEPAL, 0.1% sodium dodecyl sulfate (SDS)) containing protease inhibitors (Pierce, Rockford, IL, USA) and phosphatase inhibitors (Sigma, St. Louis, MO, USA). Protein concentration was determined using a bicinchoninic acid (BCA) assay kit (Pierce, Rockford, IL, USA) and 15 µg to 50 µg was separated on 10% Tris-glycine SDS-PAGE gels before electroblotting on nitrocellulose or polyvinylidene fluoride (PVDF) membrane (Bio-Rad, Hercules, CA, USA). Membranes were blocked in milk solution (4% nonfat milk, 20 mM Tris, 150 mM NaCl, pH 7.4) and probed with primary antibodies at 4 ${}^{\circ }$C overnight followed by HRPO-conjugated secondary antibodies (Sigma, St. Louis, MO, USA). All antibodies were diluted in blocking solution. HRPO activity was visualized with Amersham ECL Prime Western Blotting Detection Reagent (GE Healthcare Life Sciences, Pittsburgh, PA, USA) or SuperSignal West Femto Chemiluminescent Substrate (Thermo Fisher Scientific) and exposed on Bioflex Econo films (Clonex, Markham, Ontario, Canada). Image acquisition for Western blot quantification was done as previously described [68]. Briefly, film images were acquired on an AlphaImager 3400 (AlphaInnotech, San Leandro, CA, USA) under stable transillumination and fitted with CCD camera lacking automatic gain control. Final 16-bit 1392 × 1040 pixel images were corrected for shading to compensate for non-homogenous illumination and densitometry analysis was performed using the Image Studio Lite software (LI-COR, Lincoln, NE, USA).

### 4.10. Apoptotic DNA Fragmentation Analysis

One million of cells were plated on 60mm dishes the day prior to the assay. Cells were then treated with dimethyl sulfoxide (DMSO) (control) or 1 µM staurosporine (STS) (cat# ab120056, Abcam) for 2 to 24 h. At the end of the treatment, apoptotic DNA was isolated as previously described [69]. Briefly, cells were resuspended in 50 µL of lysis buffer (1% Igepal CA-630, 20 mM EDTA, and 50 mM Tris, pH 7.5) and pelleted at 1600 g for 5 min. The supernatant containing apoptotic DNA was collected and the extraction was repeated one more time. The supernatants were combined, brought to 1% SDS and treated with RNase A (final concentration 5 µg/µL) at 56 ${}^{\circ }$C for 2 h. Samples were then treated overnight with proteinase K (final concentration 2.5 µg/µL) at 37 ${}^{\circ }$C. Samples were then acidified with 3 M sodium acetate (pH 5.2) (final concentration 0.3 M) and apoptotic DNA fragments precipitated by adding 0.7 volume of isopropanol. Finally, DNA fragments were separated by electrophoresis on a 1% agarose gel containing SYBR safe (Thermo Fisher Scientific) and visualized under ultraviolet (UV) on an AlphaImager 3400.

### 4.11. Statistical Analysis

All results are presented as means ± SEM. Data were analysed with Pearson correlations tests, Student’s paired *t*-tests, or two-way mixed factorial ANOVA with post hoc Tukey tests as indicated. All statistical analysis was done using the statistical programming language R. *p*≤ 0.05 was considered as significant.

## 5. Conclusions

Pannexins constitute a more recently identified gap junction protein family. Panx2 is a particularly unique gap junction protein which predominantly has an intracellular localization. Our findings demonstrate that Panx2 localizes at ER-mitochondria contact sites in mammalian cells in vitro and in vivo and sensitizes cells to apoptotic stimuli. Contact sites between ER and mitochondria are highly conserved in terms of their morphological features but in mammalian cells, the composition of ER-mitochondria contact sites remains largely uncharacterized. Thus, we have identified a novel channel at the ER-mitochondria interface with relevance to cancer in the context of the regulation of apoptosis.

## Supplementary Materials

The following are available online at https://www.mdpi.com/2072-6694/11/3/343/s1, Figure S1: Anti-Panx2 antibodies from different species show that Panx2 localizes at ER-mitochondria contact sites, Figure S2: Panx2 co-localizes with ER markers, Figure S3: Panx2 signal does not co-localize with endosomal markers.

## Abbreviations

The following abbreviations are used in this manuscript:

CCCP	Carbonyl cyanide m-chlorophenyl hydrazone
EGFP	Enhanced green fluorescent protein
ER	Endoplasmic reticulum
IP3R	inositol 1,4,5-trisphosphate-gated receptor
MAMs	Mitochondria-associated membranes
MCS	Membrane contact sites
PB	Phosphate buffer
PBS	Phosphate buffer saline
STS	Staurosporine
