# Supplementary material for: Pannexin 2 Localizes at ER-Mitochondria Contact Sites
Source: Cancers (Basel). 2019 Mar 11;11(3):343. doi: 10.3390/cancers11030343 (PMC6468579; doi:10.3390/cancers11030343)
Supplement: Supplementary file 1 [file cancers-11-00343-s001.pdf]

Supplementary Materials

Pannexin 2 Localizes at ER-Mitochondria Contact Sites

Maxence Le Vasseur, Vincent C. Chen, Kate Huang, Wayne A. Vogl and Christian C. Naus

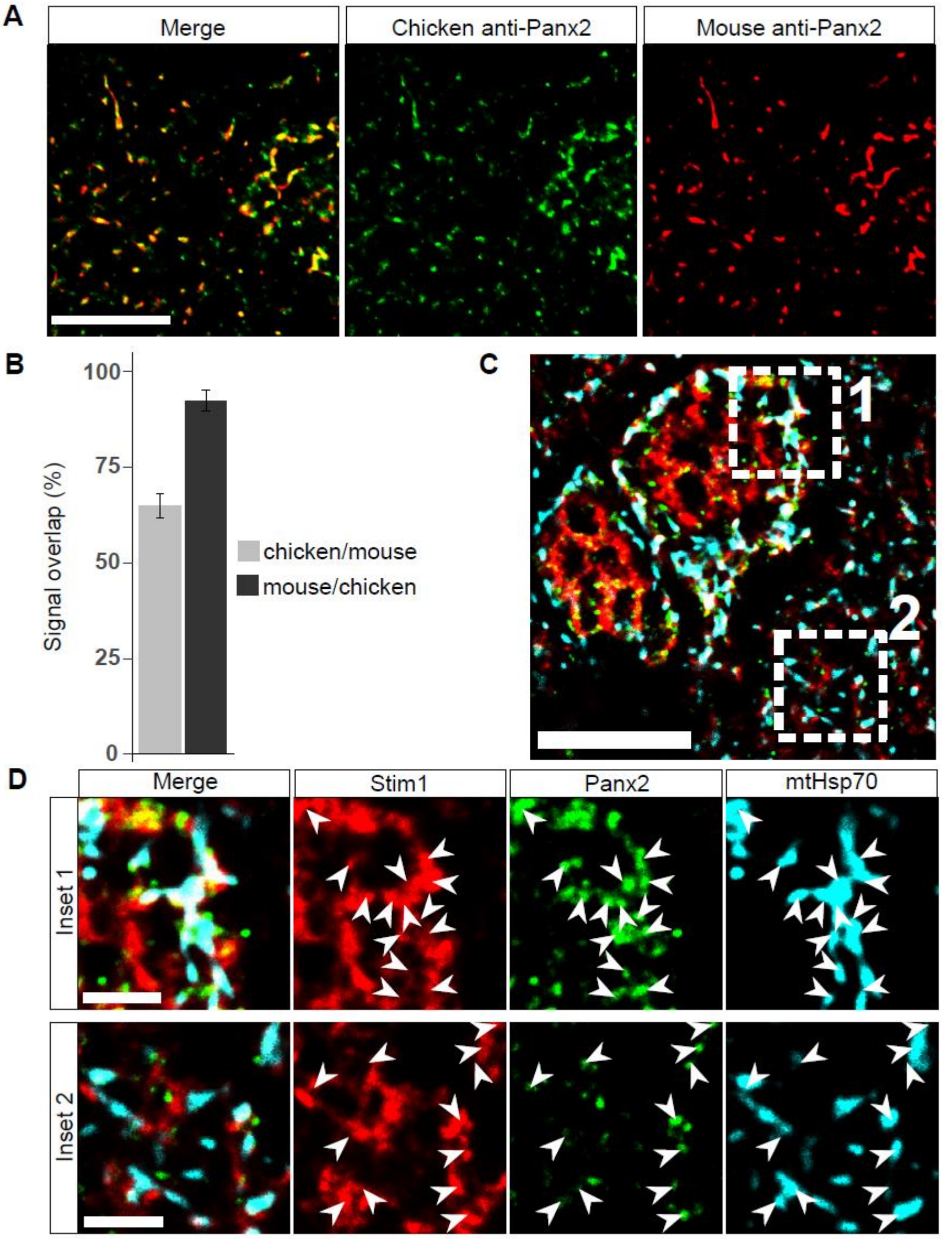

Figure S1. Anti-Panx2 antibodies from different species show that Panx2 localizes at ER-mitochondria contact sites. (**A**) Mouse brain sections immunoprobed for Panx2 using a chicken polyclonal antibody from (Diatheva, green) and a mouse monoclonal antibody (NeuroMab, red) showed overlapping staining patterns. Scale bar: 10 µm. (**B**) 64.8 ± 3.2 % of the staining obtained with the chicken anti-Panx2 antibody overlapped with the staining from the mouse primary antibody while 92.3 ± 2.7 % of the staining from the mouse antibody overlapped with the staining from the chicken antibody (n = 4). (**C**) Mouse brain sections were immunoprobed for Panx2 using the chicken anti-Panx2 antibody (green) and for Stim1 (red) and mtHsp70 (cyan) to label the ER and mitochondria respectively. Scale bar: 10 µm. (**D**) Magnification of the insets from C showing that several Panx2 punctae localize at ER-mitochondria contact sites (arrowheads). Scale bars: 2.5 µm.

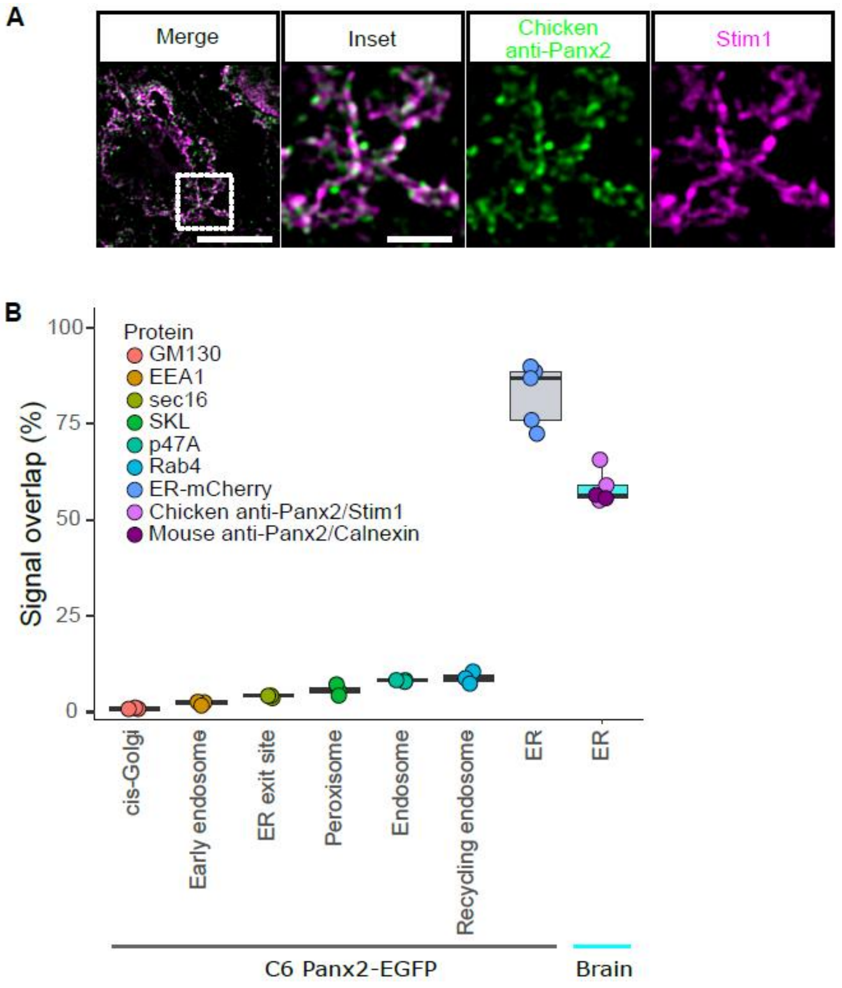

Figure S2. Panx2 co-localizes with ER markers. (**A**) Representative image of a brain section stained with a chicken anti-Panx2 antibody and an antibody against the ER protein Stim1. Endogenous Panx2 co-localized substantially with the ER marker. Scale bars: 10 µm and 2.5 µm (inset). (**B**) Co-localization between Panx2 and various organelle markers was calculated using the Manders' coefficients (expressed in percentage). A minimal coefficient value of 0 corresponds to non–overlapping images while a maximal value of 100% represents perfect overlap between both images. The low coefficient values (<10%) indicate poor co-localization between Panx2 and the secretory and endocytic organelle markers. In contrast, Panx2 co-localized strongly with ER markers in C6 Panx2-EGFP cells (82.78 ± 3.56%) or in brain sections (58.4 ± 1.94%).

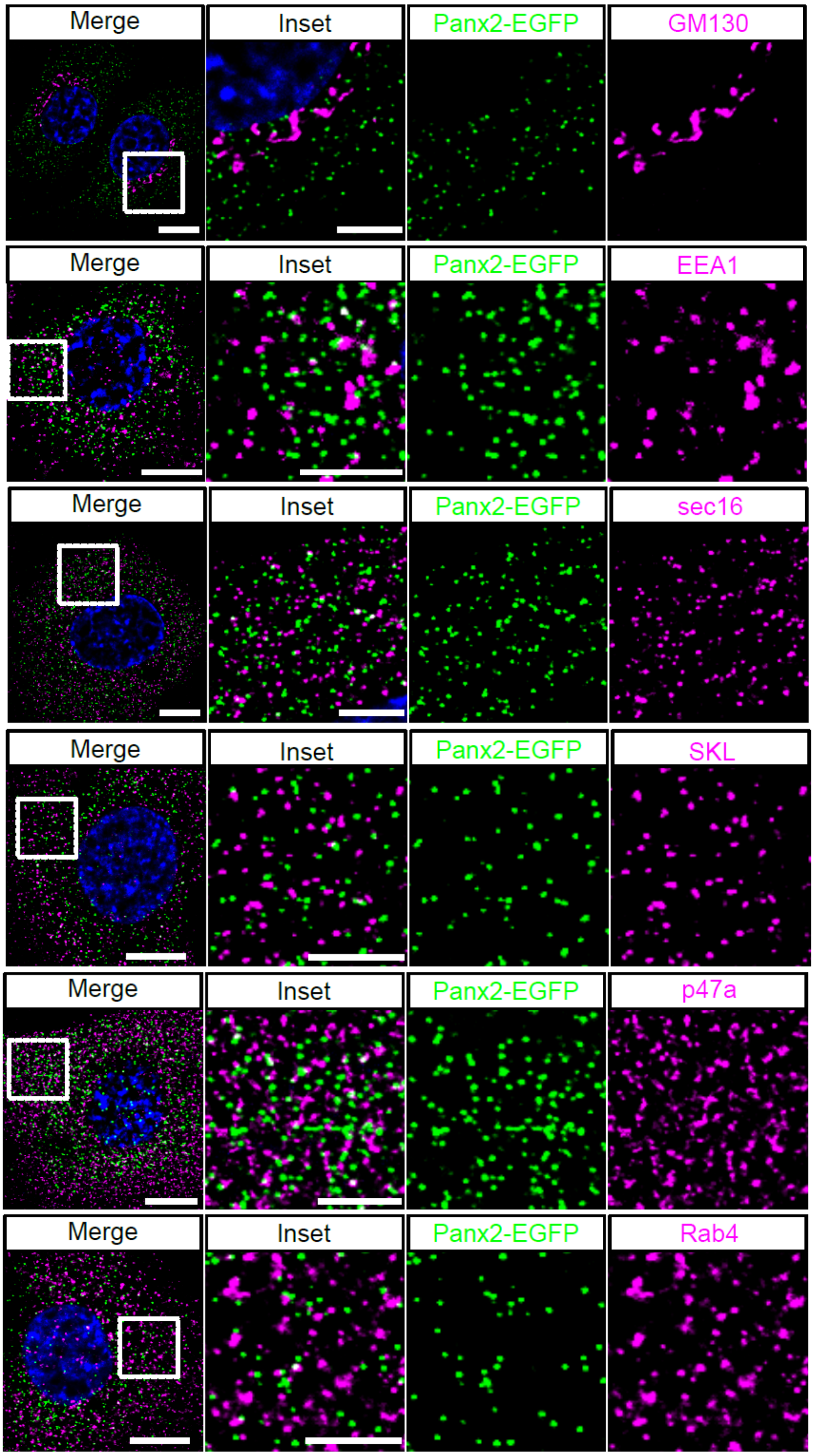

Figure S3. Panx2 signal does not co-localize with endosomal markers. Panx2–EGFP signal did not co-localize with secretory and endocytic organelle markers. The absence of co-localization with sec16 indicates that over-expressing Panx2-EGFP does not trap Panx2 at ER exit sites. Scale bars: 10 μm and 5 μm (insets).
